# Shingles in Pediatrics: A Reminder Against Age-Based Assumptions

**DOI:** 10.7759/cureus.85571

**Published:** 2025-06-08

**Authors:** Addison H Hardin, Kenneth J Adams, Rhonda Graham

**Affiliations:** 1 Research, Edward Via College of Osteopathic Medicine, Huntsville, USA; 2 Pediatrics, Kids Cove Pediatrics, Huntsville, USA

**Keywords:** herpes zoster, immunocompetent child, pediatrics, shingles, varicella vaccine, varicella zoster virus, vesicular rash

## Abstract

Herpes zoster, or shingles, is a clinical syndrome caused by the varicella-zoster virus (VZV). It is often associated with a decline in cell-mediated immunity, typically seen in older adults or individuals with compromised immune systems. Because of this association, considering a patient's age can provide valuable insight for physicians when diagnosing shingles in patients presenting with a rash. However, this focus on age may complicate diagnosis in cases where the rash occurs in individuals who do not fit the typical clinical profile, such as children. Herpes zoster is relatively uncommon in otherwise healthy children without a history of VZV infection during the first year of life or in utero. We present a case of a previously healthy, fully immunized 10-year-old girl with no significant medical history who presented with a painful vesicular rash following a dermatomal distribution on her left arm. This case serves as an important reminder not to make age-based assumptions when considering differential diagnoses.

## Introduction

Herpes zoster, commonly referred to as shingles, occurs when the varicella-zoster virus (VZV) is reactivated. The primary infection with this virus leads to varicella, also known as chickenpox. After a primary infection, VZV progresses to a latent infection with the virus either traveling retrograde through sensory axons to the dorsal root ganglia or reaching cranial and spinal ganglia through viremia. This latent infection can occur with wild-type VZV and the varicella vaccine strain [1]. Herpes zoster, or shingles, is a direct consequence of the reactivation of the latent VZV in the sensory ganglia and reactivates to form painful cutaneous blisters that can affect at least one to three dermatomes after decreased T-cell immunity [1]. Age has traditionally been an important diagnostic factor in diagnosing herpes zoster. The incidence of herpes zoster rises steeply after age 50 and is primarily a disease of adulthood [2].

Herpes zoster is also known to occur in children, although uncommon [3]. The incidence of herpes zoster in children has drastically declined over the years in the United States after the introduction of the varicella (chickenpox) vaccine in 1995, followed by the implementation of the two-dose vaccine schedule (first dose at age 12 to 15 months and second dose at age 4-6 years) in 2007 [1,3]. A population-based study found that the incidence of herpes zoster among children aged 0 to 17 years declined by 72% from 2003 to 2014, decreasing from 74 to 26 per 100,000 person-years overall. During the study period, incidence rates were 38 per 100,000 person-years among vaccinated children and 170 per 100,000 person-years among unvaccinated children [3]. Most cases of herpes zoster seen in children have a history of infection with varicella. However, herpes zoster can occur in immunized children who received the varicella vaccine, even if they have no previous exposure history. The varicella vaccine contains a live attenuated virus that can lead to latent infection, which may later reactivate to cause herpes zoster, similar to how wild-type VZV causes infection [3,4]. One study concluded that some pediatric herpes zoster cases are from the vaccine-strain VZV by obtaining specimens from the vesicles and sending them to the National VZV Laboratory at the Centers for Disease Control and Prevention for serology and PCR [4]. Although herpes zoster can be caused by the vaccine strain from the VZV vaccine, the benefits outweigh the risks, as the incidence decreases sharply compared to those who do not receive the vaccine [4]. Here, we present a rare case of herpes zoster in a 10-year-old female patient with no history of chickenpox who had received both doses of the varicella vaccine.

## Case presentation

**Figure 1:**
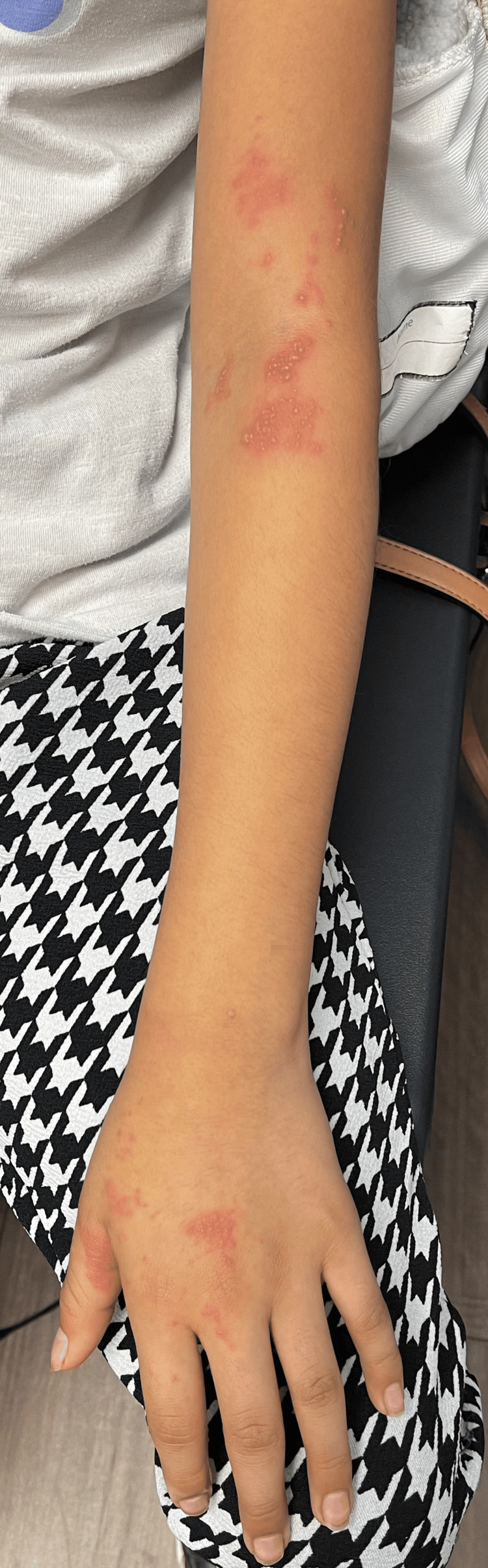
Clusters of vesicular lesions on an erythematous background along the C6 and C7 dermatomes of the left arm and hand.

**Figure 2:**
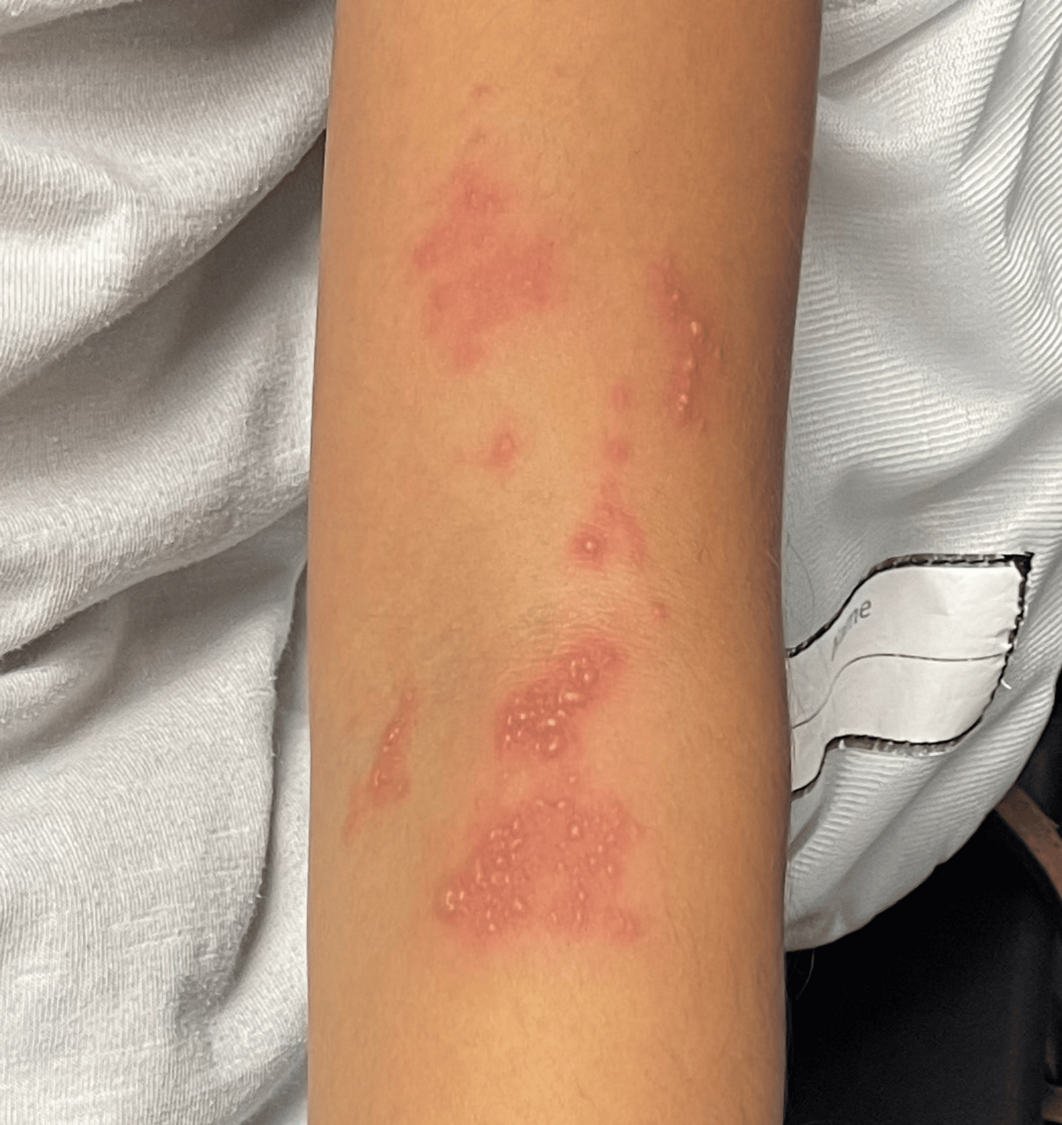
Close-up of vesicular grouping along the C6 dermatome in the left antecubital fossa, demonstrating characteristic clustered vesicles on an erythematous base.

**Figure 3:**
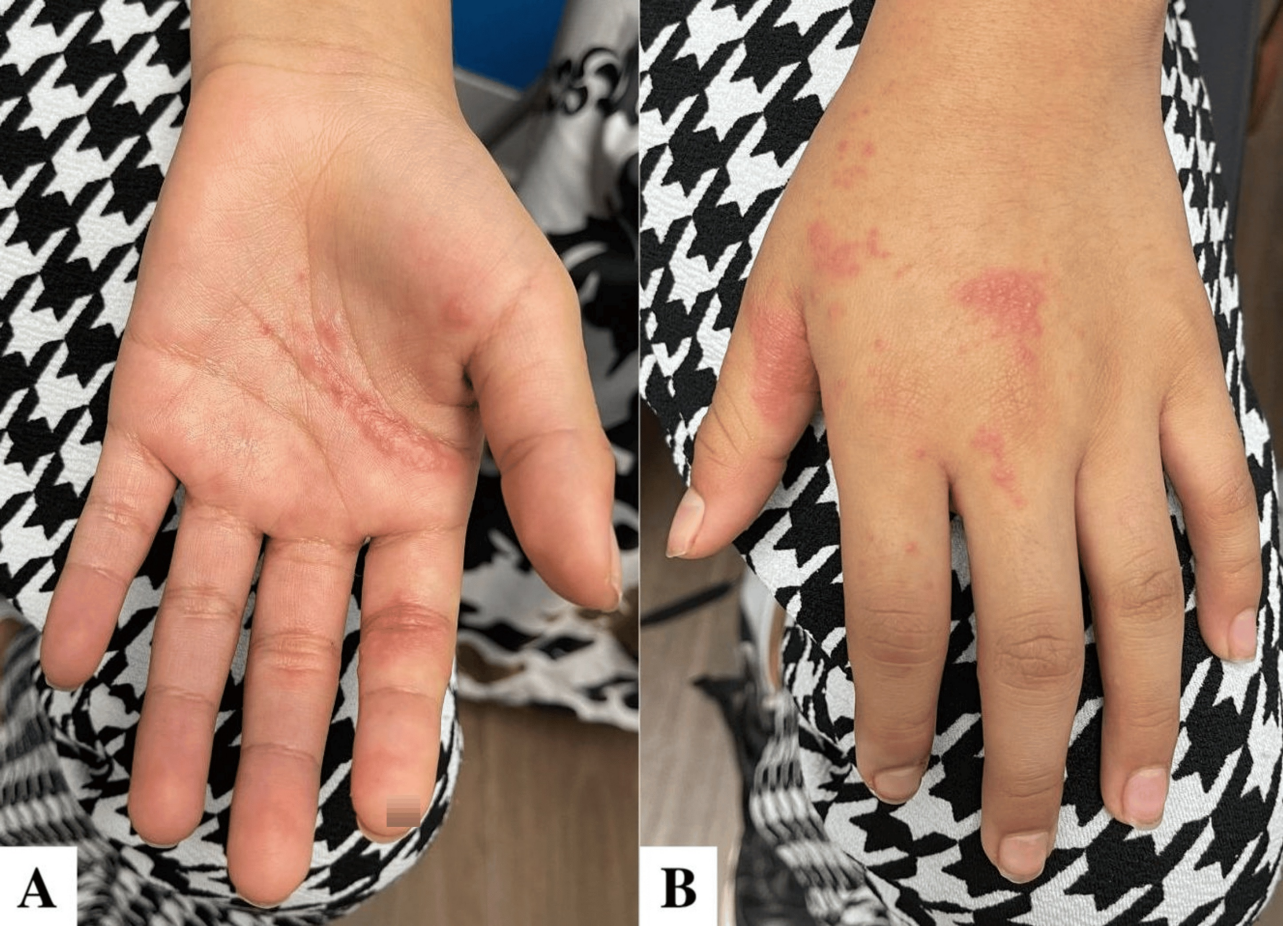
Close-up of the palmar (A) and dorsal (B) aspects of the left hand, showing clustering of vesicular lesions along the C6 and C7 dermatomes.

A 10-year-old female patient with no significant medical history presented with her mother to our pediatric clinic due to concerns about a rash on her left arm. The rash began four days prior to presentation, starting just above her left antecubital fossa, with new lesion clusters appearing on the palmar and dorsal aspect of her left hand around the base of the thumb after the initial onset. The rash began as painful, itchy, and accompanied by a burning sensation and arm stiffness. When blisters started to develop, her mother brought her in for evaluation. At the time of presentation, no treatments had been attempted at home, and the patient had no prior history of a similar rash. The patient was fully vaccinated according to the CDC-recommended schedule, including receiving separate MMR and varicella vaccines at 12 months of age, followed by the combined MMRV vaccine at the age of four years. According to her mother, the patient never had chickenpox and was not exposed to varicella in utero. The child had no significant medical history suggestive of immunocompromise, and no other family members had experienced a similar rash. During the physical examination of the patient's left arm, distinct bands of clustered vesicular lesions were observed. These lesions appeared on an erythematous background and were located specifically along the C6 and C7 dermatomes. Notably, one group of lesions was concentrated around the left antecubital fossa, while another cluster traversed the palmar creases of the left hand and extended around the base of the thumb, continuing to the dorsum of the hand (Figures 1-3). There was no evidence of pus or honey-colored crusting of note. The patient described burning pain upon palpation of the left arm lesions. Her vital signs and the remainder of the physical exam were unremarkable.

A clinical diagnosis of herpes zoster was at the top of our differential given the characteristic burning pain associated with the vesicular rash, as well as the distribution of the rash involving two dermatomes. She was given a five-day course of valacyclovir 500mg to be taken three times a day and mupirocin 2% topical ointment to apply on the rash three times daily to help prevent secondary bacterial infection. Both mother and daughter were appropriately counseled in English and Spanish regarding shingles and the possibility of spreading the virus and causing chickenpox in other individuals. They were instructed to return to the clinic for re-evaluation if no improvement was seen or the condition worsened within 72 hours of starting the medications. The patient was seen in the clinic for a follow-up two weeks later and exhibited complete resolution of her symptoms after following the treatment plan.

## Discussion

In this case, a 10-year-old female patient presented with a four-day history of a painful, erythematous, pruritic rash with vesicular lesions localized to the C6 and C7 dermatomes of her left arm and hand. Despite being fully immunized--including separate MMR and varicella vaccines at 12 months of age and the combined MMRV vaccine at four years of age--and having no history of chickenpox, the clinical features of burning pain, dermatomal distribution, and vesicular eruption strongly supported a diagnosis of herpes zoster.

Although herpes zoster is often a clinical diagnosis, several differentials were considered based on the rash’s pruritic, vesicular, and erythematous characteristics. Poison ivy dermatitis was considered but ruled out due to the absence of linear, streaked lesions typically associated with plant exposure, as well as the patient's lack of outdoor exposure. Contact dermatitis and insect bites were excluded given the clear dermatomal distribution and presence of neuropathic burning pain, which are uncharacteristic of those conditions. Impetigo was also unlikely, as there was no honey-colored crusting, a classic finding specific to this bacterial skin infection. In this case, the diagnosis of herpes zoster was made clinically without confirmatory PCR testing, consistent with current guidelines, which reserve laboratory testing for atypical presentations or cases at risk for complications [5]. 

While cases of herpes zoster in immunocompetent children without a history of varicella have been documented [6,7], this is another example of its presentation in an atypical patient population. In one detailed case report of a seven-year-old immunocompetent male patient with herpes zoster, the authors summarized previously reported pediatric cases, noting that many occurred in vaccinated children without known prior varicella infection and presented with similar dermatomal rashes and pain [7]. This reinforces the potential for herpes zoster to occur in children who appear to be low-risk and highlights the importance of maintaining clinical suspicion in atypical cases. While the introduction of the varicella vaccine in 1995 has led to a significant decline in herpes zoster cases among vaccinated children, as supported by epidemiological studies, this review emphasizes that sporadic cases can still occur, particularly in unique clinical scenarios. These findings highlight the importance of continued surveillance to monitor trends and detect rare presentations in both vaccinated and unvaccinated populations.

Despite its rarity in children, physicians must remain vigilant in recognizing herpes zoster, as delayed or missed diagnoses can lead to complications that may significantly impact patient outcomes. Herpes zoster can lead to complications, the most common of which is postherpetic neuralgia, defined as pain persisting after rash resolution or recurring months to years later [8]. While this is the most common complication in adults, this phenomenon is uncommon in children, with the estimated risk being less than 2% in patients under 60 years of age [8,9]. Secondary bacterial skin infection is the most common complication in children [9,10]. Rarely, reactivation of VZV can occur without evidence of a skin rash, leading to an atypical pain syndrome called zoster sine herpete. Diagnosis of this condition is confirmed by serologic and PCR testing demonstrating the VZV reactivation [5,9]. Herpes zoster can have more complicated disease manifestations that are less common but can occur especially in immunocompromised individuals, including ocular manifestations (herpes zoster ophthalmicus and acute retinal necrosis), disseminated zoster (with more extensive cutaneous involvement and visceral or neurologic involvement causing pneumonia, hepatitis, myelitis, meningitis or encephalitis [5,9].

The decision to treat this patient with oral antiviral and mupirocin ointment was selected to aid in symptomatic management and prevent further complications of this disease. Treatment with antiviral therapy is recommended within the first 72 hours of rash onset, but may also be used in other situations, such as a new cluster of lesions after 72 hours, as seen in this case [11]. Antiviral therapy aims to prevent new lesion formation, encourage resolution of cutaneous lesions, and decrease viral shedding [11]. The patient was counseled on personal hygiene (hand washing, covering exposed lesions) and encouraged to avoid scratching to prevent the spread of the virus to household members and secondary bacterial infection. The treatment regimen used in this case proved effective, completely resolving our patient's symptoms.

There are currently no definitive treatment guidelines for herpes zoster in children, mainly due to the rarity of the condition and limited pediatric research. As a result, management often relies on extrapolating recommendations from adult guidelines. Trusted pediatric resources like the AAP Red Book and Harriet Lane Handbook provide recommendations generally aligned with adult regimens but emphasize appropriate dosing and monitoring specific to children [9,12]. Although age has traditionally guided clinical suspicion for herpes zoster, this case, along with the case reports discussed earlier, adds to the growing evidence that immunocompetent, vaccinated children can still present with the disease outside typical age groups. This highlights the need for further research to establish evidence-based treatment guidelines tailored specifically to the pediatric population.

## Conclusions

This case emphasizes the need to maintain a high level of suspicion for herpes zoster in pediatric patients, even when typical risk factors--such as immunocompromise or a history of varicella infection--are absent. Although herpes zoster is rare in children, it is still a potential diagnosis that requires prompt recognition and management to prevent complications. By presenting this case, we aim to raise awareness of herpes zoster in atypical patient populations and highlight the importance of thorough history-taking and clinical examination. Ongoing vigilance and documentation of such cases will enhance our understanding of herpes zoster epidemiology in vaccinated children and inform future guidelines for diagnosis and management.
